# Non-Anatomic Reconstruction in Multiligament Knee Injuries: A Functional Approach

**DOI:** 10.3390/medicina61010053

**Published:** 2025-01-01

**Authors:** Mihai Hurmuz, Cătălin-Adrian Miu, Daniel Ceachir, Romulus-Fabian Tatu, Mihai Andrei, Bogdan Andor, Alexandru Catalin Motofelea, Călin Tudor Hozan

**Affiliations:** 1 Department XV, Discipline of Orthopedics, “Victor Babeș” University of Medicine and Pharmacy Timișoara, Eftimie Murgu Square 2, 300041 Timișoara, Romania; hurmuz.mihai@umft.ro (M.H.); daniel.ceachir@umft.ro (D.C.); tatu.fabian@umft.ro (R.-F.T.); 2 Orthopedics Unit, “Dr. Victor Popescu” Emergency Military Clinical Hospital, Gheorghe Lazăr Street 7, 300080 Timișoara, Romania; my.andrei8@gmail.com (M.A.); andor.bogdan@umft.ro (B.A.); 3 Center for Molecular Research in Nephrology and Vascular Disease, Discipline of Nephrology, Department VII/Internal Medicine II, Faculty of Medicine, “Victor Babeș” University of Medicine and Pharmacy, 300041 Timișoara, Romania; alexandru.motofelea@umft.ro; 4 Department of Surgical Disciplines, Faculty of Medicine and Pharmacy, University of Oradea, 410087 Oradea, Romania; chozan@didactic.uoradea.ro

**Keywords:** knee instability, ligament reconstruction, arthroscopic, open technique, IKDC Questionnaire, Lysholm Knee Scoring Scale, KOOS

## Abstract

*Background/Objectives*: Multiligament knee injuries, involving damage to multiple stabilizing structures, present a significant challenge in orthopedic surgery, often resulting in knee instability and compromised function. While anatomic ligament reconstruction has been traditionally advocated, non-anatomic techniques may provide effective alternatives, particularly for patients with moderate functional demands who do not require high-level athletic performance. *Material and methods*: In this study, we assessed the outcomes of a non-anatomic, hybrid surgical approach involving combined arthroscopic and open non-anatomic ligament reconstruction in 60 patients with multiligament knee injuries. Using simplified reconstruction methods for the medial collateral ligament (MCL) and lateral collateral ligament (LCL), we tailored the procedures to the needs of active, non-professional patients. Functional outcomes were evaluated using the International Knee Documentation Committee (IKDC) Questionnaire, Lysholm Knee Scoring Scale, and Knee Injury and Osteoarthritis Outcome Score (KOOS). *Results*: Postoperative improvements were significant, with the total IKDC score increasing from a median of 39.1 preoperatively to 75.9 postoperatively, Lysholm from 61.0 to 87.0, and KOOS from 47.6 to 85.7 (*p* < 0.01). The results demonstrated significant improvements across all scoring systems, with enhanced knee stability, reduced pain, and better quality of life. *Conclusions*: These findings support the feasibility of non-anatomic reconstructions as a practical solution for patients seeking a return to daily activities and recreational sports without the complexity of full anatomic reconstruction.

## 1. Introduction

The sensation of knee instability involves the frequent feeling of the knee “giving way” or “dislocating”, often making everyday activities such as climbing stairs and walking on uneven surfaces quite difficult. Knee dislocation, which typically results from significant trauma, is commonly accompanied by other injuries. Consequently, knee instability has a substantial impact on the quality of life of affected individuals [1]. Fortunately, there are several treatment options available to manage this issue [2]. Traditional treatments have involved addressing symptoms conservatively, surgically repairing injured ligaments, or reconstructing them using grafts from the patient’s body, a donor, or synthetic materials. More recent techniques aim to repair or replace the knee’s stabilizing structures as much as possible [3]. Both of these methods have improved outcomes for daily activities [4,5].

Advancements in technology and medical research are constantly expanding the range of treatment options for knee instability [6]. Cutting-edge techniques, including minimally invasive procedures and regenerative medicine, offer promising alternatives to traditional treatments [7,8,9]. These innovative approaches not only focus on alleviating symptoms but also on promoting tissue regeneration and enhancing long-term joint stability [10], such as combined anterior cruciate ligament (ACL)/posterior cruciate ligament (PCL) and collateral ligament reconstruction, which have shown promising results in improving knee stability and function [11]

The importance of comprehensive rehabilitation programs for individuals with knee instability cannot be overstated. These programs incorporate a combination of physical therapy, strength training, and proprioception exercises to optimize the recovery process and prevent future knee injuries [12,13,14]. Additionally, advancements in biomechanics research have led to the development of specialized braces and orthotics designed to provide enhanced support and stability [15].

This study evaluates the outcomes of combining arthroscopic anatomic cruciate ligament reconstruction with open non-anatomic collateral ligament reconstruction techniques tailored to patients with moderate functional demands. The approach focuses on simplified reconstruction methods for collateral ligaments, particularly for the medial collateral ligament (MCL) and lateral collateral ligament (LCL), while maintaining anatomic techniques for ACL and PCL reconstructions. With numerous indications and controversies surrounding combined techniques, this study aims to contribute valuable insights into optimizing surgical strategies for knee instability. With numerous indications and controversies still surrounding combined arthroscopic and open non-anatomic ligament reconstruction techniques, this study could contribute valuable insights to the literature regarding the efficacy of combining these surgical techniques.

Moreover, our objective was to observe the outcomes of non-athletes moderate active patients who underwent surgical treatment using combined arthroscopic and non-anatomic reconstructive procedures, assessing significant improvements in knee function and quality of life as measured by the IKDC Questionnaire, Lysholm Knee Scoring Scale, and KOOS.

These outcomes could guide clinicians in optimizing surgical strategies to enhance recovery and long-term knee stability while improving patient-reported quality of life.

## 2. Materials and Methods

### 2.1. Selection Criteria

This retrospective cohort study was conducted from 2019 to 2023, focusing on patients with confirmed multiligament knee injuries who received surgical treatment. The study aimed to evaluate the effectiveness of a combined arthroscopic and open non-anatomic ligament reconstruction technique using a non-anatomic approach tailored for patients with moderate activity levels.

The inclusion criteria encompassed adult patients aged 18 to 60 years with multiligament knee injuries confirmed through clinical examinations and imaging, involving at least two of the major knee ligaments (ACL, PCL, MCL, and LCL). Patients were required to present with chronic injuries (more than six weeks from the time of trauma and not more than 6 months after trauma) and to be physically active, defined as engaging in moderate levels of physical activity prior to the injury. Eligible participants needed to have sufficient overall health to undergo surgical intervention and be compliant with postoperative rehabilitation, as determined by a comprehensive preoperative evaluation. Patients with important comorbidities such as osteoporosis, cancer, autoimmune diseases, or metabolic disorders were excluded from the cohort. Individuals with associated meniscal or chondral injuries requiring repair were also included, taking into account these did not diverge from the primary focus on ligament reconstruction. All patients provided informed consent and agreed to participate in the follow-up evaluations. Patients were excluded if they had acute ligament injuries requiring immediate surgery. These criteria ensured that the sample comprised patients who could benefit from the surgical intervention, thereby enhancing the reliability of the outcomes.

The surgical procedure combined arthroscopic and open techniques, performed by a surgeon with over 10 years of experience in knee arthroscopy and sports traumatology. The arthroscopic procedures included anterior cruciate ligament (ACL) reconstruction, posterior cruciate ligament (PCL) reconstruction, combined cruciate reconstructions, and meniscal surgeries as needed, depending on the specific injury patterns observed in each patient. The average duration of the surgery was approximately 1 h and 30 min. The approach was designed to simplify the reconstruction process by using non-anatomic techniques, prioritizing functional outcomes over exact anatomical replication.

For medial collateral ligament (MCL) reconstruction, we employed two simplified methods: a single-bundle graft technique, which provided adequate valgus stability using autograft tissue anchored proximally to the femur and distally to the tibia, and the Danish technique, which utilized a minimally invasive approach with either an autograft or synthetic graft to enhance medial stability while reducing recovery time.

Lateral collateral ligament (LCL) reconstruction involved three techniques adapted based on the specific patient’s needs: the Arciero technique, which utilized an autograft passed through femoral and fibular tunnels to restore lateral stability with minimal surgical exposure; the Larson sling technique, which is also fibula-based and employed an allograft passed through a fibula tunnel and anchored at the femur, emphasizing mechanical stability; and a modified version of the LaPrade technique, where the modifications included reducing the number of surgical tunnels to minimize operative time and using a single-bundle graft instead of a double-bundle approach. These adjustments aimed to simplify the procedure while maintaining adequate lateral support and stability. The modifications were tailored to the functional demands of the patient population studied.

**Figure 1:**
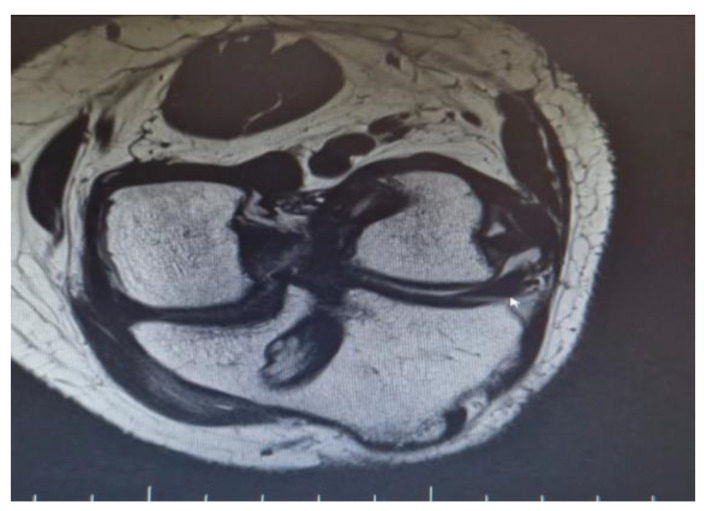
MRI demonstrating a single Achilles allograft used to reconstruct both the ACL and MCL, showing graft positioning and fixation.

**Figure 2:**
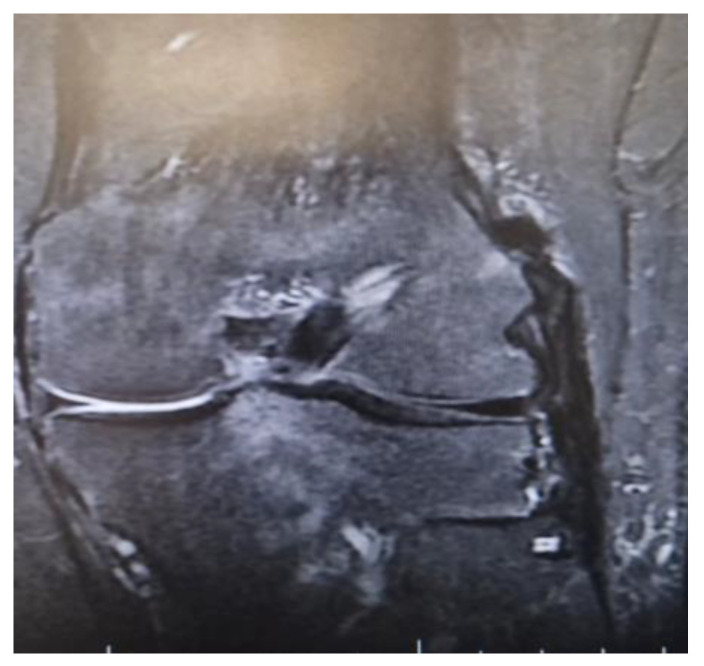
MRI demonstrating a single Achilles allograft used to reconstruct ACL, LCL, and ALL, with clear visualization of the graft distribution.

There were also a couple of cases on which a single Achilles allograft was used to reconstruct both the ACL and MCL or the ACL and LCL and ALL. In the cases where the ACL and MCL were reconstructed, the ACL was reconstructed in a standard manner, with the Achilles bone block on the femoral condyle, and after fixing the graft in the tibial tunnel, the remaining allograft was used to reconstruct the MCL. When the ACL and LCL were reconstructed with a single Achilles allograft, the femoral tunnel was created in an OUTSIDE-in technique. The ACL part was fixed with screws on the femoral tunnel and the remaining Achilles allograft was split in two, on the reconstructed LCL limb and the ALL on the other (Figure 1 and Figure 2).

Postoperative care followed a standardized rehabilitation protocol. The initial phase (0–2 weeks) focused on immobilization with a knee brace and limited weight-bearing. This was followed by progressive rehabilitation, including range-of-motion exercises and quadriceps strengthening from weeks 2 to 6. The advanced phase (from week 6 onward) incorporated proprioceptive training and sport-specific exercises, aiming for a gradual return to recreational activities.

Clinical assessments such as the Lachman test, Pivot Shift Test (PST), varus stress test (Var), and valgus stress test (Val) were included, ensuring a broader and more objective evaluation of functional outcomes (detailed in the Supplementary Materials).

Clinical outcomes were assessed using three validated scoring systems: the International Knee Documentation Committee (IKDC) Questionnaire, the Lysholm Knee Scoring Scale, and the Knee Injury and Osteoarthritis Outcome Score (KOOS). These scores are extensively utilized and validated instruments for evaluating knee function, symptoms, and patient quality of life. The KOOS is a thorough, self-administered questionnaire that assesses five domains: pain (KOSSP), symptoms (KOSSSy), activities of daily living (KOSSA), sports/recreation (KOSSp), and knee-related quality of life (KOOSQ), rendering it appropriate for monitoring outcomes in patients with knee injuries and osteoarthritis over time [16,17]. These subcategories provide a comprehensive assessment of knee functionality, symptoms, and the impact of knee conditions on daily living and quality of life. The IKDC score, created by the International Knee Documentation Committee, offers a standardized assessment of knee functionality and is commonly utilized in clinical research to evaluate the efficacy of interventions, particularly in cases of ACL and other ligament injuries [18,19]. It encompasses both subjective and objective elements to assess a patient’s perceived knee functionality in conjunction with physical examination results. The IKDC (International Knee Documentation Committee) score includes subjective and objective measures of knee function. IKDC3 evaluates the highest level of activity a patient can perform without pain, capturing the functional limitations imposed by knee instability or injury. The Lysholm score, initially developed to assess outcomes after knee ligament surgery, emphasizes eight primary symptoms, such as pain, instability, and functional impairments [20,21]. The Lysholm Knee Scoring Scale consists of eight subsections, each assessing a distinct aspect of knee health. These include the presence and severity of limping, the need for walking aids, instances of knee locking, sensations of instability or “giving way”, pain intensity during activities, post-exertion swelling, challenges in stair climbing, and the ability to perform a full squat. Each subsection contributes to the overall score, with higher scores indicating better knee functionality. For example, Lysholm 7 evaluates the patient’s ability to ascend stairs, Lysholm 4 measures knee instability, and Lysholm 5 assesses pain intensity. Collectively, these scales offer a comprehensive perspective on knee health and are essential for tracking recovery, evaluating interventions, and measuring patient satisfaction with knee-related therapies.

These tools measured improvements in knee function, pain levels, and overall quality of life. All patients provided written informed consent prior to their participation in the study. The study protocol was reviewed and approved by the institutional ethics committee, with approval reference number 20207/28 August 2024.

### 2.2. Statistical Analysis

Continuous variables following a normal distribution were presented as means with standard deviation (SD), while non-normally distributed data were presented as medians with interquartile range (IQR). The normality of the distribution was evaluated utilizing the Shapiro–Wilk test. Differences among groups for normally distributed continuous data were assessed utilizing Welch’s *t*-test for two groups or ANOVA for more than two groups. Post hoc analyses, when necessary, were conducted utilizing the Bonferroni correction to adjust for multiple comparisons. For non-normally distributed continuous data, the Mann–Whitney U test and the Wilcoxon signed-rank test were employed for two-group comparisons, while the Kruskal–Wallis test was applied for comparisons involving three or more groups. The false discovery rate was applied to adjust for multiple comparisons in the Mann–Whitney U test, Wilcoxon signed-rank test, and Kruskal–Wallis test. Categorical data were analyzed utilizing the χ^2^ test or Fisher’s exact test, particularly when expected cell counts were below five. Categorical data were reported as frequencies (*n*) and percentages (%). A priori power analysis was conducted with at least 80% statistical power with a 95% confidence interval. All statistical analyses were performed utilizing R Studio version 3.6.0 using the packages stats, dplyr, coin, multcomp, and pwr. A significant *p*-value was considered <0.05.

## 3. Results

The study comprised 60 participants with a median age of 38 years (IQR 32–44), of whom 87% were male (*n* = 52) and 13% were female (*n* = 8). Participants predominantly lived in urban regions (60%) as opposed to rural regions (40%). The majority of subjects participated in physical therapy (88%), while a lesser percentage received a combination of physical therapy and hydrotherapy (12%). The median rehabilitation duration was 38 days (IQR 24–40), and the median symptom duration before intervention was 12 months (IQR 8–24), as observed in Table 1.

The surgical procedure combined arthroscopic and open techniques. The combinations of procedures included ACL + MCL reconstructions (*n* = 28), ACL + LCL reconstructions (*n* = 20), and cases involving all four ligaments (*n* = 25). Specifically, there were four cases of ACL + PCL reconstructions and eight cases of ACL + PCL + MCL reconstructions These combinations were tailored to the specific injury patterns and functional demands of the patients.

The majority of individuals were employed in sedentary positions (67%), while 25% participated in moderate physical work, and merely 8.3% engaged in strenuous physical labor. Educational qualifications differed, with 43% holding a university degree and 40% having finished high school. The median BMI was documented at 29.4 (IQR 24.8–30.0), signifying a predominantly overweight population. ACL and LCL reconstructions were performed in five patients (8.3%), highlighting the relatively low prevalence of injuries involving these two ligaments. Similarly, ACL and MCL reconstructions were performed in eight patients (13%), indicating a moderate frequency for this combination. The most common reconstructions were ACL with LCL, seen in 15 cases (25%), and ACL with MCL, observed in 20 cases (33%), suggesting that these combinations account for the majority of ligament injuries requiring surgical intervention.

Less frequently, ACL and PCL reconstructions were performed in four patients (6.7%), reflecting a lower occurrence of simultaneous anterior and posterior cruciate ligament injuries. More complex reconstructions, such as ACL, PCL, and MCL, were required in six patients (10%). The rarest combination, involving PCL, ACL, and MCL, was performed in only two patients (3.3%).

These findings suggest that while injuries combining ACL with either LCL or MCL dominate, more complex patterns requiring triple ligament reconstructions are considerably less common. This distribution provides valuable insights into injury prevalence and may help guide clinical decisions and surgical planning.

### Functional Outcomes: Preoperative Versus Postoperative

The comparative analysis of preoperative and postoperative conditions utilizing diverse clinical scores revealed significant enhancements post-intervention, as seen in Table 2. All components of the International Knee Documentation Committee (IKDC) scores exhibited significant changes, with *p*-values consistently below 0.013, indicating substantial enhancements in knee functionality. For instance, IKDC1 exhibited an elevation from a preoperative median of 1.0 to a postoperative range of 2.0–4.0, indicating improved knee function and stability. IKDC6 specifically assesses locking or catching symptoms of the knee. In this study, there was no statistically significant change observed in IKDC6 scores, indicating that these symptoms were less responsive to the intervention. The overall IKDC score demonstrated a significant increase, rising from a preoperative median of 32.6–45.2 to a postoperative range of 69.5–95.4 (F-statistic = 67.64, *p* < 0.013). These results highlight a substantial improvement in knee functionality, probably due to the intervention.

The Lysholm Knee Scoring Scale demonstrated statistically significant enhancements in most items postoperatively (*p* < 0.01) (Table 3), signifying diminished pain and enhanced knee function. LYSHOLM7 exhibited an elevation in scores from a preoperative median range of 0.0–6.0 to a postoperative range of 9.7–10.0 (*p* < 0.013), indicating diminished symptoms and improved knee stability. The total Lysholm score exhibited substantial improvement, increasing from a preoperative range of 48.0–73.0 to a postoperative range of 73.6–93.2 (*p* < 0.01). The increase in scores signifies substantial postoperative enhancements, indicating improved knee functionality and alleviation of symptoms.

The KOOS demonstrated significant postoperative enhancements across various subdomains (Table 4). KOOSP1, which evaluates symptoms and pain, diminished from a preoperative range of 2.0–3.0 to a postoperative range of 0.0–3.0 (*p* < 0.01), indicating a reduction in pain levels. Correspondingly, KOOSA1, indicative of daily living activities, decreased from a preoperative median of 1.0 to 0.0 postoperatively (*p* < 0.013), underscoring improved functionality in executing routine tasks. The total KOOS score significantly increased, from a preoperative median of 42.9–67.3 to a postoperative range of 63.1–89.3 (*p* < 0.013), indicating substantial enhancements in knee symptoms, pain alleviation, and quality of life following the intervention.

Although the majority of items in the IKDC, Lysholm, and KOOS scales demonstrated substantial enhancements, a few anomalies were identified. For example, LYSHOLM6 (swelling) and KOOSP8 exhibited no notable alterations, with *p*-values exceeding the significance threshold. Notwithstanding these exceptions, the majority of measures in this study achieved statistical significance, underscoring the intervention’s efficacy.

The analysis of preoperative and postoperative results indicates significant enhancements across various metrics post-intervention. The findings from Table 3 indicate substantial postoperative enhancements in knee function, pain alleviation, and daily functionality across all three assessment instruments.

All subdomains of the IKDC scores, with the exception of IKDC6 (*p* = 0.08), demonstrated statistically significant improvements (*p* < 0.01). IKDC1 exhibited an enhancement from a preoperative median of 1.0 to a postoperative range of 2.0–4.0 (*p* < 0.01). This trend persisted across additional subdomains, notably IKDC3, which rose from a median of 1.0 (4.0, 5.0) preoperatively to 8.0 (9.0, 10.0) postoperatively (*p* < 0.01), signifying substantial improvements in knee function and stability. The cumulative IKDC score highlights these advancements, increasing from a preoperative median of 31.0 (39.1, 44.1) to 69.5 (75.9, 95.4) postoperatively, with a *p* < 0.01, thereby affirming a comprehensive improvement in knee health following the intervention.

The study findings demonstrate significant enhancements in knee function, pain alleviation, and overall quality of life post-intervention, evaluated through the IKDC, Lysholm, and KOOS scoring systems. Postoperative scores significantly improved across all three tools when compared to preoperative scores, with the majority of *p*-values falling below 0.01, underscoring the intervention’s effectiveness.

## 4. Discussion

This study’s findings indicate that patients who undergo surgical treatment using a combined arthroscopic and open non-anatomic ligament reconstruction technique have markedly enhanced knee function, alleviated pain, and improved participants’ quality of life, as evidenced by elevated scores on the IKDC, Lysholm, and KOOS scales.

When comparing ACL + ALL reconstruction to ACL + MCL or ACL + LCL reconstructions, prior research suggests that different ligament combinations address distinct instability patterns. For instance, Sonnery-Cottet et al. (2015) documented significant improvements in knee stability and function following simultaneous ACL and anterolateral ligament (ALL) reconstruction [22], as evidenced by enhancements in the Lysholm, IKDC, and pivot shift scores [11]. Similarly, studies by Noyes et al. [23] reported that ACL + MCL reconstructions are particularly effective in managing valgus instability, while ACL + LCL reconstructions are necessary for addressing varus instability and postero-lateral corner deficiencies. Furthermore, a systematic review and meta-analysis by Lima et al. (2021) found that combined ACL and ALL reconstruction resulted in better postoperative clinical outcomes compared to isolated ACL reconstruction, particularly in reducing residual pivot shift and rerupture rates [24].

However, this study’s emphasis on non-anatomic reconstruction highlights a critical distinction from anatomic techniques. While anatomic reconstructions aim to replicate native ligament biomechanics, non-anatomic methods prioritize functional stability and operative simplicity. This approach is particularly advantageous for patients who do not require high-level athletic performance, as it minimizes surgical time and postoperative rehabilitation challenges while delivering comparable functional outcomes.

In comparison to anatomic collateral ligament reconstructions, non-anatomic approaches, as employed in this study, simplify surgical techniques and reduce operative time while still yielding comparable functional outcomes [23]. For example, Ardern et al. found that anatomic ACL + MCL reconstructions resulted in slightly better stability metrics but required longer rehabilitation durations and were associated with higher surgical complexity. Our findings align with these studies, as the combination of ACL + non-anatomic collateral ligament reconstructions demonstrated significant improvements in IKDC, Lysholm, and KOOS scores [25]. The choice of ligament combinations should be tailored to the patient’s specific instability patterns, activity level, and functional demands and allograft availability.

In orthopedic settings, quality-of-life assessments are often underemphasized compared to other medical fields.

The significance of the IKDC score as a measure of knee functionality and quality of life is thoroughly documented in the literature. Williams et al. (2020) emphasized that elevated IKDC scores following ACL reconstruction were associated with enhanced quality of life, reinforcing the notion that IKDC is a reliable metric for assessing the impact of surgical procedures on knee health and patient-reported outcomes [10]. Our study similarly demonstrated an elevation in the total IKDC score from a median of 31.0 to 69.5, indicating enhancements in both knee stability and overall quality of life for patients following surgery.

A systematic review by Filbay et al. (2022) affirmed that untreated or inadequately treated ACL and meniscal injuries can adversely affect long-term quality of life, physical activity, and economic productivity [1]. Our findings substantiate the effectiveness of prompt and thorough surgical intervention, as postoperative KOOS scores in our study demonstrated improved quality of life and diminished symptoms and pain. This underscores the essential function of comprehensive surgical methods, such as combined ligament reconstruction, in alleviating the detrimental impacts of knee instability on daily activities and quality of life.

Furthermore, Yanardag et al. (2021) discovered that pain, balance, and gait function substantially influence the quality of life in individuals suffering from knee and hip pain [4]. This finding corresponds with our study’s results in the Lysholm and KOOS scores, where we noted significant enhancements in symptoms and pain levels postoperatively, indicating that diminished pain and enhanced stability can markedly influence patient-reported quality of life. The Lysholm score notably improved from a median of 48.0 preoperatively to 73.6 postoperatively, demonstrating that surgical intervention not only restores physical knee function but also mitigates pain, thereby enhancing patients’ quality of life.

Innovative therapies for knee instability are progressing, as demonstrated by Körner et al. (2020), who highlighted the advantages of integrating advanced rehabilitation protocols with surgical intervention [5]. In our study, patients underwent a combination of physical therapy and, for some, hydrotherapy postoperatively, which likely facilitated the notable enhancements in functionality and pain levels. This corresponds with Körner’s findings, highlighting the significance of a multidisciplinary approach in improving postoperative recovery and ensuring long-term joint stability. Our findings emphasize the importance of optimizing postoperative management strategies, such as physical therapy and hydrotherapy, in facilitating recovery and improving functional outcomes. While pharmacological interventions, such as NSAIDs, may play a role in other clinical settings, this study focused exclusively on physical rehabilitation as the primary modality for postoperative care.

By combining these treatment modalities with proper nutrition and lifestyle modifications, individuals with knee instability can experience a significant improvement in their overall quality of life. It is crucial to note that a multidisciplinary approach is of great importance in effectively managing knee instability [26,27].

This study illustrates notable enhancements in knee functionality and quality of life after combined ligament reconstruction.

## 5. Limitations

This study has certain limitations that should be acknowledged. The predominance of male participants (87%) restricts the ability to evaluate gender differences in outcomes, and future research should address this imbalance to ensure broader generalizability. Additionally, the relatively small sample size and short-term follow-up period limit the scope of our findings. Expanding the cohort to include more diverse populations and conducting studies with longer follow-up durations will be critical for validating these results. Moreover, while we focused on non-anatomic reconstruction within a single cohort, the absence of a control group prevents direct comparisons with anatomic reconstruction techniques. Future studies should incorporate comparative analyses to provide more comprehensive insights. Future studies with extended follow-ups (5–10 years or longer) will be critical to assessing the durability of joint stability, functional outcomes, and the potential onset of osteoarthritis. Notwithstanding these constraints, the findings align with the current literature supporting comprehensive surgical and rehabilitation strategies for knee injuries [4,6,12]. Furthermore, reliance on subjective outcome measures, such as IKDC, Lysholm, and KOOS scores, highlights the need for additional objective assessments, such as biomechanical evaluations and imaging studies, to enhance the robustness of the findings [18,19].

## 6. Conclusions

This study indicates that the combination of arthroscopic and open non-anatomic ligament reconstruction markedly enhances knee function, alleviates pain, and improves the quality of life for individuals with knee instability.

## Figures and Tables

**Table 1:** Demographic and clinical characteristics of the study population.

Characteristic	*n* = 60
Age	38 (32, 44)
Gender	
Female	8 (13%)
Male	52 (87%)
BMI (kg/m^2^)	29.4 (24.8, 30.0)
Place of residence	
Rural	24 (40%)
Urban	36 (60%)
Type of rehabilitation	
Physical therapy (PT)	53 (88%)
PT + Hydrotherapy	7 (12%)
Duration of rehabilitation (weeks)	38 (24, 40)
Duration of symptoms (months)	12 (8, 24)
Type of work	
Intensive physical work	5 (8.3%)
Moderate physical work	15 (25%)
Sedentary work	40 (67%)
Educational level	
High school	24 (40%)
Post-secondary	7 (12%)
Vocational school	3 (5.0%)
University	26 (43%)
Combinations of reconstructions	
ACL + LCL (single Achilles allograft)	5 (8.3%)
ACL + MCL (single Achilles allograft)	8 (13%)
ACL + LCL (ACL autograft + hamstring allograft)	15 (25%)
ACL + MCL (BTB allograft for ACL and hamstring autograft for MCL)	20 (33%)
ACL + PCL (ACL autograft + PCL Achilles allograft)	4 (6.7%)
ACL + PCL + MCL	6 (10%)
PLC + ACL + MCL (single Achilles allograft for ACL and PLC + hamstring autograft for MCL)	2 (3.3%)

Data are presented as median (interquartile range) for continuous variables and frequency (percentage) for categorical variables. BMI: Body Mass Index; ACL: Anterior Cruciate Ligament; LCL: Lateral Collateral Ligament; MCL: Medial Collateral Ligament; PCL: Posterior Cruciate Ligament; PLC Postero-Lateral Corner; PT: Physical Therapy.

**Table 2:** Preoperative and postoperative IKDC scores.

	*n*	Preoperatory Status	Postoperatory Status	Test Statistic
		(*n* = 30)	(*n* = 30)	
IKDC1	60	1.0 1.0 1.0	2.0 2.5 4.0	F_1,58_ = 33.43, *p* < 0.01 ^1^
IKDC2	60	0.0 4.0 5.0	8.0 9.0 9.1	F_1,58_ = 44.06, *p* < 0.01 ^1^
IKDC3	60	1.0 4.0 5.0	8.0 9.0 10.0	F_1,58_ = 57.70, *p* < 0.01 ^1^
IKDC4	60	1.0 2.0 2.0	2.0 3.0 3.1	F_1,58_ = 18.42, *p* < 0.01 ^1^
IKDC5	60	1.0 1.0 2.0	1.0 2.5 4.0	F_1,58_ = 18.13, *p* < 0.01 ^1^
IKDC6	60	0.0 1.0 1.0	1.0 1.0 1.0	F_1,58_ = 3.13, *p* = 0.08 ^1^
IKDC7	60	0.0 1.0 2.0	2.0 3.0 3.1	F_1,58_ = 45.71, *p* < 0.01 ^1^
IKDC8	60	1.0 1.0 1.0	2.0 2.5 4.0	F_1,58_ = 26.19, *p* < 0.01 ^1^
IKDC9	60	12.0 14.5 17.2	25.0 30.0 33.2	F_1,58_ = 60.03, *p* < 0.01 ^1^
IKDC10	60	2.0 4.0 5.0	7.9 8.0 10.0	F_1,58_ = 29.31, *p* < 0.01 ^1^
Total IKDC	60	31.0 39.1 44.1	69.5 75.9 95.4	F_1,58_ = 70.66, *p* < 0.01 ^1^

IKDC: International Knee Documentation Committee score. Data are presented as median (25th percentile, 75th percentile). ^1.^ Wilcoxon Signed-Rank Test.

**Table 3:** Preoperative and postoperative Lysholm scores.

	*n*	Preoperatory Status	Postoperatory Status	Test Statistic
		(*n* = 30)	(*n* = 30)	
LYSHOLM1	60	3.0 3.0 3.0	3.0 5.0 5.0	F_1,58_ = 19.96, *p* < 0.01 ^1^
LYSHOLM2	60	0.0 2.0 5.0	5.0 5.0 5.0	F_1,58_ = 39.21, *p* < 0.01 ^1^
LYSHOLM3	60	9.7 10.0 15.0	15.0 15.0 15.0	F_1,58_ = 13.11, *p* < 0.01 ^1^
LYSHOLM4	60	10.0 15.0 20.0	20.0 20.0 25.0	F_1,58_ = 29.30, *p* < 0.01 ^1^
LYSHOLM5	60	10.0 15.0 20.0	19.6 20.0 25.0	F_1,58_ = 7.50, *p* = 0.01 ^1^
LYSHOLM6	60	0.0 6.0 10.0	6.0 6.0 10.0	F_1,58_ = 0.08, *p* = 0.78 ^1^
LYSHOLM7	60	0.0 2.0 6.0	9.7 10.0 10.0	F_1,58_ = 41.39, *p* < 0.01 ^1^
LYSHOLM8	60	0.9 1.0 4.0	4.0 4.5 5.0	F_1,58_ = 20.44, *p* < 0.01 ^1^
Total Lysholm	60	48.0 61.0 73.0	73.6 87.0 93.2	F_1,58_ = 33.53, *p* < 0.01 ^1^

Lysholm: Lysholm Knee Scoring Scale. Data are presented as median (25th percentile, 75th percentile). ^1.^ Wilcoxon Signed-Rank Test.

**Table 4:** Preoperative and postoperative KOSS scores.

	*n*	Preoperatory Status	Postoperatory Status	Test Statistic
		(*n* = 30)	(*n* = 30)	
KOOSP1	60	2.0 3.0 3.0	0.0 1.0 3.0	F_1,58_ = 12.80, *p* < 0.01 ^1^
KOOSP2	60	2.0 3.0 3.0	0.0 0.0 1.1	F_1,58_ = 27.93, *p* < 0.01 ^1^
KOOSP3	60	1.9 3.0 3.0	0.0 0.0 1.0	F_1,58_ = 36.77, *p* < 0.01 ^1^
KOOSP4	60	1.0 2.0 3.0	0.0 0.0 1.0	F_1,58_ = 17.27, *p* < 0.01 ^1^
KOOSP5	60	0.0 2.0 3.0	0.0 0.0 0.0	F_1,58_ = 38.50, *p* < 0.01 ^1^
KOOSP6	60	1.9 3.0 3.0	0.0 0.0 2.0	F_1,58_ = 41.11, *p* < 0.01 ^1^
KOOSP7	60	0.0 1.0 3.0	0.0 0.0 1.0	F_1,58_ = 5.28, *p* = 0.03 ^1^
KOOSP8	60	0.0 0.0 2.0	0.0 0.0 1.0	F_1,58_ = 3.18, *p* = 0.08 ^1^
KOOSP9	60	0.0 2.0 2.0	0.0 0.0 1.0	F_1,58_ = 10.40, *p* < 0.01 ^1^
KOOSSy1	60	0.0 2.0 3.0	0.9 1.0 1.0	F_1,58_ = 8.52, *p* < 0.01 ^1^
KOOSSy2	60	1.0 1.0 3.0	0.0 0.0 1.0	F_1,58_ = 13.53, *p* < 0.01 ^1^
KOOSSy3	60	1.0 2.0 3.0	0.0 1.5 2.0	F_1,58_ = 4.24, *p* = 0.04 ^1^
KOOSSy4	60	0.9 1.0 3.0	0.0 0.0 1.0	F_1,58_ = 17.24, *p* < 0.01 ^1^
KOOSSy5	60	0.9 1.0 1.2	0.0 3.5 4.0	F_1,58_ = 2.74, *p* = 0.10 ^1^
KOOSSy6	60	0.0 1.0 2.0	1.0 3.0 4.0	F_1,58_ = 13.59, *p* < 0.01 ^1^
KOOSA1	60	1.0 2.0 2.0	0.0 0.0 1.0	F_1,58_ = 60.41, *p* < 0.01 ^1^
KOOSA2	60	1.0 1.5 3.0	0.0 0.0 1.0	F_1,58_ = 38.56, *p* < 0.01 ^1^
KOOSA3	60	0.0 1.0 2.0	0.0 0.0 1.0	F_1,58_ = 3.95, *p* = 0.05 ^1^
KOOSA4	60	1.0 1.0 3.0	0.0 0.0 1.0	F_1,58_ = 23.17, *p* < 0.01 ^1^
KOOSA5	60	0.0 2.0 2.0	0.0 0.0 2.0	F_1,58_ = 14.86, *p* < 0.01 ^1^
KOOSA6	60	1.0 1.0 1.1	0.0 0.0 0.0	F_1,58_ = 24.65, *p* < 0.01 ^1^
KOOSA7	60	1.0 2.0 2.0	0.0 0.0 0.0	F_1,58_ = 135.79, *p* < 0.01 ^1^
KOOSA8	60	1.0 1.0 2.0	0.0 0.0 0.0	F_1,58_ = 30.10, *p* < 0.01 ^1^
KOOSA9	60	0.9 1.0 2.0	0.0 0.0 0.0	F_1,58_ = 21.88, *p* < 0.01 ^1^
KOOSA10	60	1.0 2.0 2.0	0.0 0.0 0.0	F_1,58_ = 50.83, *p* < 0.01 ^1^
KOOSA11	60	1.0 1.0 2.0	0.0 0.0 0.1	F_1,58_ = 41.07, *p* < 0.01 ^1^
KOOSA12	60	1.0 1.0 2.0	0.0 0.0 0.1	F_1,58_ = 41.97, *p* < 0.01 ^1^
KOOSA13	60	1.0 1.0 2.0	0.0 0.0 0.1	F_1,58_ = 54.22, *p* < 0.01 ^1^
KOOSA14	60	0.9 1.0 2.0	0.0 0.0 1.0	F_1,58_ = 7.94, *p* = 0.01 ^1^
KOOSA15	60	0.9 1.0 2.0	0.0 0.0 1.0	F_1,58_ = 25.85, *p* < 0.01 ^1^
KOOSA16	60	1.0 2.0 4.0	0.0 1.0 2.0	F_1,58_ = 18.62, *p* < 0.01 ^1^
KOOSA17	60	1.0 1.0 2.1	0.0 0.0 1.0	F_1,58_ = 21.55, *p* < 0.01 ^1^
KOOSSp1	59	2.0 3.0 3.0	0.0 0.5 1.0	F_1,57_ = 76.20, *p* < 0.01 ^1^
KOOSSp2	60	2.0 3.0 3.0	0.0 1.0 1.2	F_1,58_ = 25.21, *p* < 0.01 ^1^
KOOSSp3	60	2.9 3.0 4.0	1.0 1.0 1.1	F_1,58_ = 50.60, *p* < 0.01 ^1^
KOOSSp4	60	3.0 4.0 4.0	0.0 1.0 2.0	F_1,58_ = 72.36, *p* < 0.01 ^1^
KOOSSp5	60	2.0 3.0 4.0	0.0 1.0 2.0	F_1,58_ = 25.41, *p* < 0.01 ^1^
KOOSQ1	60	3.0 4.0 4.0	1.0 3.0 4.0	F_1,58_ = 11.63, *p* < 0.01 ^1^
KOOSQ2	60	2.9 4.0 4.0	1.0 2.0 3.0	F_1,58_ = 23.79, *p* < 0.01 ^1^
KOOSQ3	60	3.0 4.0 4.0	0.9 1.0 2.0	F_1,58_ = 45.72, *p* < 0.01 ^1^
KOOSQ4	60	2.0 3.0 4.0	1.0 1.0 2.0	F_1,58_ = 25.53, *p* < 0.01 ^1^
KooS total	60	42.9 47.6 65.5	63.1 85.7 89.3	F_1,58_ = 55.92, *p* < 0.01 ^1^

Data are presented as median (25th percentile, 75th percentile), ^1.^ Wilcoxon Signed-Rank Test.

## Data Availability

The data supporting the results of this study are available from the corresponding author upon reasonable request.

## Supplementary Materials

The following supporting information can be downloaded at: https://www.mdpi.com/article/10.3390/medicina61010053/s1, Details of the clinical assessments (Lachman, PST, Var, and Val tests) alongside objective MRI findings to provide additional clarity and transparency.
